# The Weak Link: Hypotonia in Infancy and Autism Early Identification

**DOI:** 10.3389/fneur.2021.612674

**Published:** 2021-02-04

**Authors:** Lidia V. Gabis, Meirav Shaham, Odelia Leon Attia, Shahar Shefer, Ruth Rosenan, Tal Gabis, Michal Daloya

**Affiliations:** ^1^Weinberg Child Development Center at Safra Children's Hospital, Sheba Medical Center, Ramat Gan, Israel; ^2^Sackler Faculty of Medicine at Tel Aviv University, Tel Aviv, Israel; ^3^Department of Statistics, University of Haifa, Haifa, Israel; ^4^Faculty of Medicine, Charles University, Prague, Czechia

**Keywords:** autism, infant, hypotonia, comorbidity, girls

## Abstract

**Background:** Presenting symptoms and age specific differential diagnosis of Autism Spectrum Disorder (ASD), determine the age of initial assessment and the age of a definite diagnosis. The AAP recommends screening all children for ASD at 18 and 24 months followed by a comprehensive evaluation for children with developmental concerns. More recently it has been recommended that the evaluation should be performed at a younger age, with a diagnosis being made as early as the beginning of the second year of life resulting in earlier intensive intervention.

**Objective:** To assess early developmental milestones in a cohort of children diagnosed with Autism Spectrum Disorder (ASD) in order to find an objective and reliable early marker. We suggest that low muscle tone- hypotonia, is a sign that meets the above criteria of consistency and reliability and may be related to early diagnosis.

**Methods:** We compared age distributions of ASD diagnosis in the presence of hypotonia in a dataset of 5,205 children diagnosed at Keshet Center. One thousand, one hundred eighty-two children (953 males) were diagnosed with ASD and compared to other developmental diagnoses. Within the ASD cohort we further analyzed for gender and pre-maturity differences.

**Results:** In the presence of hypotonia, the mean age for ASD diagnosis was significantly lower (by 1.5 years for males and females) and this effect increased in children born at term as compared to pre-maturity.

**Conclusions:** Hypotonia is a recognizable marker of ASD and may serve as a “red flag” to prompt earlier recognition and neurodevelopmental evaluation toward an autism diagnosis.

## Introduction

The diagnosis of Autism Spectrum Disorder (ASD) is clinical. Presenting symptoms and age specific differential diagnosis determine the age of initial assessment and the age of a definite diagnosis. The American Academy of Pediatrics recommends screening all children for ASD at 18 and 24 months followed by a comprehensive evaluation for children with developmental concerns (1). However, more recently it has been recommended that the evaluation should be performed at a younger age, with a diagnosis being made as early as the beginning of the second year of life (2).

When a child receives a final diagnosis of ASD, an intervention program including intensive approach and parental guidance is implemented (3). Early intervention is paramount to improve the function and social participation of children with ASD (4). As such, an accurate identification of easy to recognize, measurable and reliable “red flags” is essential to improve outcomes in autism.

Since motor milestones are easy to recognize and measure, we suggest that low muscle tone- hypotonia, is a reliable early “red flag” to prompt ASD evaluation that could translate into an earlier diagnosis, intervention and possibly an improved outcome.

Early intervention is an umbrella term covering many different services funded by a variety of public and private sources. Available services are determined by each locality. Public Law 99-457, 1986 that was reauthorized in 1991 as PL 102-119 led to expanded services for young children with disabilities. Part C of the Individuals with Disabilities Act (IDEA) has assisted in developing comprehensive services that mandate a family directed approach (5). The main message that health-care providers convey to parents is that an early diagnosis warrants early professional services that are designed to promote the child's communicative, behavioral and social functioning development as well as assist him/her in acquiring better adaptive skills. An earlier ASD diagnosis prompts an earlier intervention, which will result in more effective improvements in the child's functioning (6). However, at times, an early diagnosis of ASD is made based on obvious and significant developmental deficits that are associated with more severe autism. In such cases, early diagnosis does not always assure a good prognosis (4).

Early signs and symptoms that can be recognized during the first year of life: By 4 months of age, babies should not only be crying but using other means of communicating their needs such as vocalizations and facial expressions (7, 8). Lack of evolution of body language and lack of modulation of eye contact should raise concerns. During the first 6 months, babies increase their motor control by incorporating movements to express their needs. Before learning how to crawl they reach the motor milestone of “working toward an object” by moving their bodies and limbs toward people and objects of interest. As infants learn about the reactions of others, they reach the social milestone of raising both arms in a request to be picked up (9). Infants that present with motor gaps such as head lag, low muscle tone (hypotonia), exaggerated or lack of response to sensory stimuli (such as noise or touch), should raise concerns and elicit a more extensive neurological evaluation (10, 11). Additional hints may present as overt motor asymmetries that do not improve with time. Minor inconsistent asymmetry involving asynchronous movements of limbs in infants is part of normal development. Consistent asymmetry should prompt a more extensive evaluation because movements should be synchronous until about age two with the development of the dominant hand (4, 12).

From 6 to 12 months of development motor control advances along with the emergence of a more extensive vocal repertoire such as razzing and babbling. The motor pathway is an indicator of maturation and it may serve as a sign for normal general developmental processes (11).

Following the rapid growth of non-verbal communication during the second year of life, the repertoire of motor gestures such as pointing, waving, nodding, clapping and more, should increase spontaneously after 1 year of age (13).

With the increase in motor activity and control, unusual behaviors may become more obvious. For example, hand flapping, walking in circles, lining objects, and a particular interest in spinning objects may be reported by parents early on. Unusual early motor movement patterns are common in infants that are subsequently diagnosed with ASD and may be an early sign of atypical behaviors (14–16). These patterns could be consistent asymmetric movements or milestones appearing earlier than expected, such as rolling from their belly onto their back. Other early signs include unusual motor interests such as holding a metal object instead of the usual transitional nappies and prolonged interest in mechanical objects such as spinning wheels (17).

### Hypotonia

Hypotonia is defined as decreased muscle tone or floppiness with varying degrees of progression. It occurs in multiple neuromuscular, metabolic and genetic disorders and can be a sign of global developmental delay, that may pre-dispose to a cognitive disability (18).

The severity and progression of hypotonia varies with each child and with each diagnosis. For example, children with Down syndrome or with hypotonic cerebral palsy have non-progressive low tone hypotonia while those with neuromuscular disorders such as muscular dystrophy have progressive hypotonia that worsens with time. Hypotonia present in pre-mature infants may improve with maturity of the central nervous system or evolve to cerebral palsy (19).

Benign congenital hypotonia (BCH) is a diagnosis of exclusion, given to many children after workup has been exhausted. BCH is considered a non-progressive neuromuscular disorder that does not worsen but tends to improve with time and intervention. It may have a high familial incidence that may indicate BCH is of an autosomal dominant, genetic origin (20).

Hypotonic children may also have very flexible joints either in BCH or in syndromes presenting with hypotonia such as Down syndrome and Fragile X (21). Since the infant has poor head and axial control, this combination is associated with motor delay characterized by delayed sitting and late independent walking (22).

### Hypotonia, Feeding, and Additional Influences on Motor Postural Control

Hypotonia may involve axial tone including neck muscles and the muscles around the mouth, influencing the infant's sucking and feeding abilities (21).

Positioning of the infant for feeding is a particular challenge for parents of hypotonic babies, as the child lacks head and chest control. These infants experience sucking, chewing and swallowing difficulties along with persistent drooling from the mouth. Posture control during feeding or breastfeeding may also influence eye contact and communication with the caregiver. In retrospect, feeding difficulties are common in children subsequently diagnosed with autism and may persist for a long time (23).

Hypotonia may start prenatally, and the abnormal postures can lead to a neck deformity called torticollis, that develops in some children who hold their head to one side (20, 24).

Hypotonia may be associated with global developmental delay, either as a cause or a result of delayed milestones (21, 25).

Since hypotonia, hyperlaxity and motor delay can impair an infant's ability to explore his or her environment, the infant could ignore critical visual cues resulting in impaired learning and cognitive development (26).

Additional cues to atypical development in infancy are general movements of the infant and sleep- arousal patterns. General movements are a distinct movement repertoire carried out spontaneously without external stimulation and are seen in fetuses of 9 weeks gestational age until 21 weeks post-term. General movements are helpful in the early diagnosis of an impaired central nervous system such as the specific prediction of cerebral palsy (27) and they reflect impairments of brain areas involved in cognitive development (28). Measurements of GM and sleep are particularly important to assess in infants born prematurely.

### Emphasis on Motor Development as a Key to Early Diagnosis

There is still a significant gap between “state of the art” research on autism and common practice as they relate to age of diagnosis. This gap varies in its magnitude between countries, among communities, and in relation to socioeconomic status. There is an established direct connection between early diagnosis, early intervention and subsequent outcomes, however, the path is not linear. Early diagnosis of ASD is made based on obvious and significant developmental deficits that may be associated with more severe autism, or the result of extremely observant parents. In the former, an early diagnosis does not always assure a good prognosis. The earlier the intervention, the more effective it is in improving functioning (6).

As such, accurate identification of easy to recognize, measurable and reliable “red flags” is paramount to improve outcomes in autism. We suggest that low muscle tone- hypotonia, is a sign that meets the above criteria of consistency and reliability and may indeed serve as an early “red flag” to prompt neurodevelopmental evaluation and autism screening.

We also postulate that early hypotonia may be present before birth, which can lead to the complicated deliveries, cesarean sections or general anesthesia that has been associated with Autism spectrum disorder (29–31).

## Materials and Methods

### Participants

The present study is a dataset of 5,205 children (male = 3,346, female = 1,859) out of which 1,182 were children diagnosed with ASD with documented age of ASD diagnosis (male = 953, 81% of total ASD population, female = 229, 19% of total ASD population). Among 4,023 non-ASD children there were 2,393 (59%) males and 1,630 (41%) females. These differences of sex distribution reflect the male to female ratio of ASD as compared to other diagnoses. The participants included children from January 2010 to December 2018 who underwent a diagnostic process consisting of a neurodevelopmental and psychological assessment based on DSM-IV-TR or DSM-5 criteria (32, 33). Evaluations were performed at Keshet Center- a specialized, hospital-based tertiary developmental center.

### Procedure and Measures

The study was approved by the hospital's IRB as part of a larger study predicting developmental disability including autism and intellectual level of children that were referred for a developmental evaluation (Helsinki approval 8458-11-SMC).

The clinical diagnostic process at Keshet Center is performed according to the national MOH guidelines (34). It includes a physical, neurological and a developmental exam performed by a physician specialized in pediatric neurology with special expertise in neurodevelopmental disabilities. Each child was additionally evaluated by a developmental or rehabilitation psychologist according to age. The Autism Diagnostic Observation Schedule—ADOS (35) was used to confirm the diagnoses. The physician also determined a Developmental Quotient (DQ) score based on the Denver Developmental Screening Test (DDST II) (36) up to age five, and performed a Clinical Adaptive Test/Clinical Linguistic and Auditory Milestone Scale (CAT/CLAMS) test up to age 3 years (37), in parallel to the formal psychological evaluation. Children with motor, fine motor and language delays additionally underwent a thorough evaluation for each of these areas, as performed by physical, occupational and language pathologists. In addition to the ASD diagnosis and the neurodevelopmental evaluation, all participants underwent standardized cognitive testing as part of their clinical evaluation. Specific instruments selected for cognitive testing were chosen according to the child's age and functioning level. Instruments used included the Bayley II scales (38), Mullen Scales for Early Learning (39), and cognitive tests: Stanford-Binet Fourth Edition (40), Wechsler Pre-school and Primary Scale for Intelligence—Third Edition (41), Wechsler Intelligence Scale for Children—Revised (42) Leiter-R (43) and Kaufman Brief Intelligence-Test (K-BIT) (44).

### Statistical Analyses

We explored the relationship between developmental and motor comorbidities associated with ASD and their potential effect on the age of ASD diagnosis. Age distribution of initial ASD diagnosis was divided into age sub-groups. By using parametric and non-parametric multiple comparisons that incorporated intervening factors such as gender and pre-maturity, we identified a group of comorbidities (CM) that were consistently associated with a lower age of initial ASD diagnosis. Specifically, we made comparisons of ASD diagnostic age distributions and tested for significance in the presence of low muscle-tone indicators (hypotonia, torticollis, feeding issues) and other common co-morbidities (CM) groups (motor and global delays, Developmental Coordination Disorder, speech, and language difficulties).

Since a child may have more than one CM, it is more than likely that the overall number of subjects in all CM groups is higher than the actual cohort. In this kind of stacked data structure, it is more challenging to detect differences between subgroups, as each single data point (each individual) may be shared by more than one CM and therefore has the potential to expand over a wider age range.

Gender is a significant variable that may influence diagnosis and may present with different CM between males and females (45). We analyzed gender influences on age of diagnosis and on comorbidity.

Since motor delays including hypotonia are prevalent in infants born prematurely, and given the elevated rate of autism diagnosis linked to pre-maturity (46), we further analyzed variability in age of diagnosis as related to pre-maturity.

#### Tests Used

##### Parametric tests

Pearson Chi-Square: establish correlation and significance for the presence or absence of certain CMs with ASD.

ANOVA (analysis of variance): establish significant differences between certain CMs and ASD in the age of ASD diagnosis.

##### Non-parametric tests

Wilcoxon each pair comparisons (multiple comparisons)—compare between ages of diagnosis of ASD for each of the CMs.

Kolmagorov-Smirnov: compare between distributions of ages of ASD diagnosis at the presence or absence of a certain CM.

Gender and pre-maturity in the above tests were considered as intervening factors.

## Results

The initial cohort of 5,205 children comprised of 3,346 males and 1,859 females of which there were 1,476 children with ASD, 1,200 males with ASD and 276 females with ASD. Data that included age of initial diagnosis was available in 1,182 children with ASD.

### Age of Initial Diagnosis

The ASD cohort of 1,182 children was comprised of 953 males and 229 females diagnosed initially between the age of 10 months and 12 years (*M* = 4.3 years, SD = 2.6). The age of ASD diagnosis was significantly different by gender with females being diagnosed at a younger age: males' mean age of ASD diagnosis was 4.4 years (±2.6 SD), while the mean age of ASD diagnosis for females was 3.8 years (±2.5 SD), [F_(1,1,181)_ = 10.28, *p* < 0.01]. The distribution of the ages of ASD diagnosis can be adequately described as a normal 3-mixture (M1 = 2.5, SD1 = 0.9 Pi1 = 0.50, M2 = 5.4. SD2 = 1.7, Pi2 = 0.42, M3 = 10.1, SD = 3 = 1.1, Pi3 = 0.08).

In order to address age specific comorbidities (CM), we divided the cohort to 3 sub-groups of ages, following the three means described in the 3-mixture distribution:

Category ≤2.5 years, *n* = 345, Males = 261, Females = 84 (24%)

Category ≤5.4 years, *n* = 504, Males = 404, Females = 97 (19%)

Category >5.4 years, *n* = 336, Males = 288, Females = 48 (14%)

Although the mixture proportion for age on the 2.5 years mean was the largest (50%), when we cut the categories by the mean values, the comparison became more strict, as it reduced the range of ages in that group (it will now encompass 30% of the overall population).

Of all the participants in the cohort diagnosed at the Keshet Center, there were multiple developmental diagnoses such as developmental speech and language delay, motor delay or disability, Global Developmental Delay (GDD), ADHD etc. All diagnoses present in more than 5 children in the cohort were listed in Table 1. The classification of primary vs. secondary diagnosis depended on if it was reached before or after ASD diagnosis. When a diagnosis such as GDD, motor delay or ADHD were present in a child before their ASD diagnosis, the diagnosis was considered primary, but when it occurred in a child with known ASD it was considered to be a CM or a secondary diagnosis.

**Table 1:** Comorbidities of the entire cohort by gender.

**Comorbidities**	**Total**	**Males**		**Females**	
		**Non-ASD**	**ASD**	**Non-ASD**	**ASD**
Developmental speech or language disorder	1,481	849	169	425	38
ADHD	1,403	703	388	252	60
Global delay (GD)	1,294	481	331	370	112
Behavioral/emotional	870	522	84	243	21
Intellectual disability (ID)	521	162	204	108	47
Fine motor difficulties	365	238	47	77	3
Communication	341	138	127	43	33
Hypotonia	333	148	23	155	7
Motor delay	243	150	6	87	0
Developmental coordination disorder (DCD)	206	105	50	46	5
Learning disability	206	102	41	55	8
Cerebral palsy (CP)	201	90	10	92	9
Motor impairment	163	92	19	48	4
Disorders of muscles/tendons (includes hypertonus, torticollis etc.)	155	85	4	63	3
Epilepsy	152	66	34	36	16
Feeding and eating disorders	75	35	9	28	3
Sensory motor integration difficulties	75	47	8	18	2
Anxiety	63	31	17	10	5
Sleep disorders	54	19	13	20	2
Stereotypic/involuntary movements	46	27	8	8	3

Within the ASD group, the stacked dataset included numerous CM diagnoses such as 177 children with over 5 diagnoses, 748 children with 2–4 diagnoses and 257 children with only an ASD diagnosis without any co-morbidities. For the purpose of investigating motor delays with respect to the age of ASD diagnosis, we decided to exclude children with severe physical co-morbidities, such as cardiac, gastrointestinal or other systemic disorders. We focused only on participants with neurodevelopmental and neuro- behavioral CM diagnoses which resulted in a reduced cohort size of 1,182 children. In order to further reduce the CM variability, we analyzed only CM that occurred in more than five children, other than those with hypertonus and torticollis who were included even if sparse, due to their connection to motor development.

Table 2 below describes the percentages of co-morbidities within each category of group of means.

**Table 2:** Comorbidities (CM) by age group of diagnosis.

**CM subgroups**	***N* % in sub group ≤2.5 Y**	**% in sub group ≤2.5 Y**	***N* in sub group ≤5.4 Y**	**% in sub group ≤5.4 Y**	***N* in sub group >5.4 Y**	**% in sub group >5.4 Y**
Hypotonia	17	58.6%	7	24.1%	5	17.2%
Global delay (GD)	236	51.5%	178	38.9%	44	9.6%
Hypertonus	2	50.0%	2	50.0%	0	0.0%
Sleep disorders	5	35.7%	7	50.0%	2	14.3%
Feeding and eating disorders	4	33.3%	8	66.7%	0	0.0%
Sensory motor integration difficulties	3	30.0%	6	60.0%	1	10.0%
ASD	371	29.0%	550	43.00%	358	28.0%
Motor impairment	5	26.3%	11	57.90%	3	15.8%
Communication	31	25.0%	56	45.20%	37	29.8%
Stereotypic or involuntary movements/tics	2	22.2%	4	44.4%	3	33.3%
Developmental speech and language disorders	47	21.4%	111	50.5%	62	28.2%
Anxiety	4	21.1%	7	36.8%	8	42.1%
Epilepsy	9	18.4%	23	46.9%	17	34.7%
Emotional problems	11	15.5%	30	42.3%	30	42.3%
Fine motor difficulties	6	14.6%	19	46.3%	16	39.0%
Behavioral problems	4	13.3%	16	53.3%	10	33.3%
ADHD	74	13.3%	241	43.3%	242	43.4%
Intellectual disability (ID)	28	11.8%	111	46.8%	98	41.4%
Learning disability	4	11.1%	9	25.0%	23	63.9%
Motor delay	1	11.1%	6	66.7%	2	22.2%
Developmental coordination disorder (DCD)	2	3.8%	16	30.2%	35	66.0%
Cerebral palsy (CP)	1	3.1%	14	43.8%	17	53.1%
Torticollis	0	0.0%	3	100.0%	0	0.0%

When analyzing frequent occurring CM in the early diagnosis group (<30 months) the most frequent CM were: hypotonia, global delay (GD), sleep disturbances, hypertonus, feeding and eating issues.

**Figure 1:**
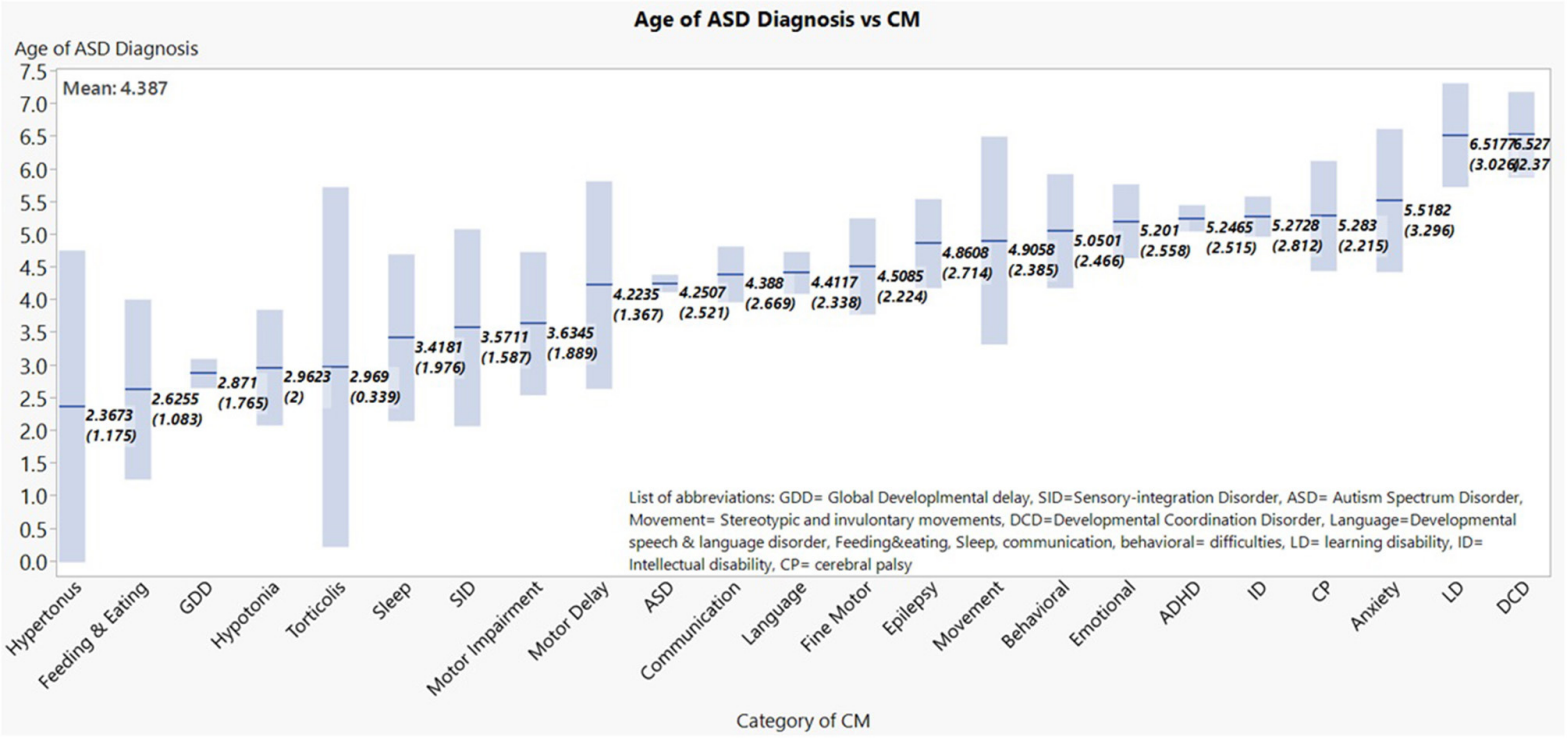
Mean age of ASD diagnosis age by respective comorbitidy sub-group are shown in ascending order, with their respective 95% confidence levels. *The number of children in the graph is higher than the total number of children with ASD since each child may get more than one diagnosis.

Within the ASD cohort, mean values of the age of ASD diagnosis were graded lowest to highest amongst CM sub-groups (Figure 1 shows the means and the 95% confidence interval of the mean for each diagnosis category). When CM were attached to age of diagnosis of ASD, the main CM that correlated with lower age of diagnosis were: GDD, hypotonia, hypertonus, torticollis, and feeding/eating disorder.

Within the subset cohort of ASD there were 29 children with hypotonia and 1,153 children without hypotonia and this diagnosis was the most frequent among early diagnosed comorbidities.

In view of the CM grouping according to age of ASD diagnosis, we examined the center's complete cohort of children for specificity and sensitivity of hypotonia and GDD diagnoses to ASD. Of the initial cohort of 5,205 children (3,346 males and 1,859 females) there were 1,476 children diagnosed with ASD (1,200 Males and 276 females). Of this population there were 303 children without ASD with hypotonia as a primary diagnosis, and 52 children with ASD and hypotonia.

Performing a Pearson chi square-test we did not find a significant difference for the ASD proportion in the presence of hypotonia (Pearson, chi square = 65.55, *p* < 0.001, for non-hypotonic children). Testing with gender as an intervening effect yielded similar results.

We further examined the proportion of children with GDD and hypotonia with or without ASD in the full cohort. There were 443 children with GDD and ASD, and 851 children with GDD without an ASD diagnosis. There were 125 children with both hypotonia and GDD, while the number of children with GDD without hypotonia was 1,169 and children without GDD nor hypotonia were 3,703.

When examining GDD, we found that GDD correlated significantly with ASD (Pearson chi square-test 29.28, p<.001), and with hypotonia (Pearson chi square-test 30.61, *p* < 0.001).

### Differences of Age of ASD Diagnosis Among Children With Hypotonia and Other Co-Morbidities

Comparisons of means using the Wilcoxon method for each pair between hypotonia and all other typical categories including a standalone ASD diagnosis, which showed that the only means differing non-significantly from hypotonia were GD, sleep, torticollis, hypertonus and feeding (hypotonia and sleep *Z* = 1.36, *p* = 0.17, torticollis with hypotonia *Z* = 1.26 *p* = 0.21, hypotonia and GDD *Z* = −0.38, *p* = 0.71, hypotonia, feeding, and eating *Z* = −0.33, *p* = 0.74, hypotonia and hypertonus *Z* = 0.03, *p* = 0.98). Hypotonia incidence was significantly linked to an early diagnosis when compared to all other CM. Therefore, an ASD early age of diagnosis cluster can be regarded with the above CMs: GD, sleep, feeding, torticollis, and hypertonus.

Over 50% of GM CM occurred more frequently within the ≤2.5Y ASD diagnosis age group, therefore we further explored the differences between GD and the remaining CMs. We observed that GM was not significantly different than hypotonia CM as it was associated with a diagnosis below 30 months. GD bared no significant differences to torticollis, hypotonia and hypertonus; thus forming a cluster of early ASD diagnosis indicators (GDD and sleep *Z* = 1.35 *p* = 0.18, GDD and torticollis *Z* = 0.91 *p* = 0.36, GDD and hypertonus *Z* = −0.39 *p* = 0.70, GDD and hypotonia *Z* = −0.38 *p* = 0.71 GDD, feeding, and eating *Z* = −0.09 *p* = 0.93).

Since hypotonia meets the criteria of a simple and relatively objective symptom, we analyzed it as a standalone CM.

In the presence of hypotonia the ASD initial diagnosis was significantly at lower age by nearly 1.5 Y in average [with hypotonia *M* = 2.96 years, SD = 2.0, without hypotonia *M* = 4.41 years, SD = 2.56, ANOVA result is F ratio (1, 3,405) = 9.23, *p* < 0.01]. Testing while assuming a non-parametric distribution resulted in similar conclusions (Kolmogorov-test, KSa = 2.48, *p* < 0.001).

#### Gender Differences

Testing for differences for age of ASD diagnosis in males as compared to females with vs. without hypotonia, resulted in significant differences for all between w/wo hypotonia groups without interaction between gender and hypotonia: Males with hypotonia *M* = 2.95 years, SD = 1.87, Males wo hypotonia *M* = 4.49 years, SD = 2.54, Females with hypotonia, *M* = 3.01 years, SD = 2.54, Females wo hypotonia *M* = 4.07 years, SD = 2.59. When testing separately using the Kolmogorov-Smirnov asymptotic test for gender w/wo hypotonia we found a higher significance for males in the presence of hypotonia vs. its absence when compared to females (Males KSa = 2.28 *p* < 0.001, Females KSa = 1.39, *p* < 0.05 calculated).

### Influence of Pre-maturity

The age of ASD diagnosis in pre-term and in term children differed significantly with pre-term ASD being identified almost 1 year earlier than children born at term: Mean diagnostic age at term = 4.3 years, SD = 2.6, *N* = 1,127 mean diagnosis at pre-term = 3.5 years SD = 2.2, *N* = 55, using the Kolmogorov-Smirnov asymptotic-test—Ksa = 1.38, *p* < 0.05.

When testing within gender groups for pre vs. in term children we found that the age of ASD diagnosis for males born at pre-term was significantly lower than those of term males. For females the age of diagnosis was similar between pre-term and term:

In Term males age of diagnosis = 4.4 years SD = 2.6, *N* = 915, Pre-term males = 3.3 years, SD = 2.2, *N* = 38, Kolmogorov-Smirnov Ksa = 1.59, *p* < 0.01, for females Ksa = 0.63, *p* > 0.05.

Testing for differences in age of ASD diagnosis in the presence or absence of hypotonia when comparing by gestational age resulted in a lower age of diagnosis in the presence of hypotonia for all children, term and pre-term: pre-term with hypotonia *M* = 2.2 years, SD = 1.1 *N* = 4, pre-term without hypotonia, *M* = 3.5 years, SD = 2.2 *N* = 55, term with hypotonia *M* = 3.1 years, SD = 2.1, term without hypotonia *M* = 4.3, SD = 2.5 pre-maturity).

Testing the effect of hypotonia separately within in term vs. pre-term cohorts using the Kolmogorv-Smirnov asymptotic-test we found that for in term children the effect is highly significant (in term KSa = 2.35, *p* < 0.001) and for pre-term children it is not significant (pre-term Ksa = 0.73, *p* > 0.05).

## Discussion

When analyzing a large cohort of more than 5,000 children diagnosed at one tertiary center, more than a quarter of participants received a diagnosis of ASD. The male gender was more prevalent in the ASD group and the common diagnoses were delays in specific developmental areas such as motor and language as well as global developmental delay (GDD) and ADHD.

When assessing the age of diagnosis, we found a large spectrum ranging from <1 year to 12 years. Clearly there are significant differences expected between children diagnosed very early on such as below 2 years with children diagnosed in late childhood. This resulted in the emergence of three age groups of children according to the age range of their initial diagnosis. Though not significant, more girls were identified younger than in the older age group.

When additional developmental diagnoses occurred in conjunction with ASD, we considered those diagnoses as comorbidities. Most children with ASD had multiple CM, while only 21% had a diagnosis of ASD without additional diagnoses (47). If the same child had different comorbidities at different ages, we accounted for the age of diagnosis of their comorbidity. We found that hypotonia was detected more frequently in the younger group, making it a good marker for an earlier ASD diagnosis. In addition, other motor difficulties such as hypertonus and torticollis also occurred more frequently in the younger group, as well as eating and feeding problems. More than half of the group diagnosed with ASD below the age of 30 months had each of the motor diagnoses and one third had eating and feeding CM. Non-significant differences were localized around hypotonia, feeding, hypertonus, and torticollis, thus, forming a cluster of indicators that may characterize an early ASD diagnosis.

All of the additional CM that occurred early, may be related to abnormal motor development such as feeding which directly relates to neck and facial musculature (48, 49).

With respect to the first aim- we indeed proved that low muscle tone is a recognizable marker of ASD and its effect on lower age of diagnosis differs according to gender with a more accentuated influence on younger boys. Hypotonia in males can accelerate the age of ASD diagnosis by an average of 1.5 Y while for females, it will be accelerated by an average of 1 Y. Since motor difficulties and ASD diagnosis are prevalent in infants born prematurely and those infants are followed prospectively from birth, the mean age of ASD diagnosis was significantly lower in pre-mature children by almost 1 year, but only in males. An ASD diagnosis in females born pre-term did not differ from term girls, probably since pre-term females display “masking” signs such as common comorbidities that result in a delayed diagnosis (48).

The effect of hypotonia was not significant within the pre-mature cohort, probably due to cohort size differences, or the myriad of common comorbidities present in pre-mature infants (46).

The sample size including hypotonia is a limitation of the study, nevertheless it was sufficient and proven significant in the various statistical tests performed.

## Data Availability Statement

The raw data supporting the conclusions of this article will be made available by the authors, without undue reservation.

## Ethics Statement

The studies involving human participants were reviewed and approved by Sheba Tel-Hashomer Hospital's IRB. Helsinki Approval 8458-11-SMC. The patients/participants provided their written informed consent to participate in this study.

## Author Contributions

LG: conceptualization, methodology, validation, writing—original draft preparation, writing—review and editing, supervision, and approval of final version. MD: writing—review and editing, supervision, and approval of final version. TG: data entry and approval of final version. RR: data entry, writing—original draft preparation, and approval of final version. SS: resources, writing—original draft preparation, project administration including ethics (IRB), and approval of final version. OL: methodology, data entry, data analysis, resources, writing—original draft preparation, and approval of final version. MS: methodology, software, data analysis, validation, writing—original draft preparation, and approval of final version. All authors contributed to the article and approved the submitted version.

## Conflict of Interest

The authors declare that the research was conducted in the absence of any commercial or financial relationships that could be construed as a potential conflict of interest.
